# A case of Epstein Barr virus-associated primary squamous cell carcinoma of stomach

**DOI:** 10.1186/s40792-021-01301-9

**Published:** 2021-11-15

**Authors:** Yuki Katsura, Takehiro Okabayashi, Kazuhiro Ozaki, Yuichi Shibuya, Jun Iwata

**Affiliations:** 1 Department of Gastroenterological Surgery, Kochi Health Sciences Center, 2125-1 Ike, Kochi-City, Kochi 781-8555 Japan; 2 Department of Diagnostic Pathology, Kochi Health Sciences Center, 2125-1 Ike, Kochi-City, Kochi 781-8555 Japan

**Keywords:** Squamous cell carcinoma, Stomach neoplasms, Epstein Barr virus

## Abstract

**Background:**

Primary squamous cell carcinoma (SCC) of stomach is extremely rare. The pathogenesis of SCC of stomach remains unclear. There is only one report that Epstein Barr virus (EBV) infection may be involved in the pathogenesis of SCC arising in the stomach ever before. Here, we report a case of Epstein Barr virus infection-associated primary SCC of stomach in a 70-year-old woman. She was presented to the referring hospital with hematemesis. Initial endoscopy revealed a bleeding gastric ulcer in the upper part of gastric corpus and the coagulation therapy was followed. After a 3-month follow-up, endoscopy revealed a submucosal tumor-like protrusion instead of an ulcer. Computed tomography revealed a mass in the upper part of stomach and swollen lymph nodes along with the lesser curvature and para-aortic lymph node. Biopsy could not confirm the definitive diagnosis. We performed total gastrectomy with para-aortic lymph node sampling. Histological analysis revealed squamous cell carcinoma with EBV infection with lymph node metastases. Tumor cells were positive for EBV-encoded small RNA (EBER) by in situ hybridization. The postoperative course was uneventful and the patient was discharged on day 11 after the operation. CapeOX was started as adjuvant chemotherapy, and the patient remains alive without recurrence 7 months after surgery.

**Conclusion:**

This is the first case report of EBV infection-associated primary SCC of the stomach diagnosed by in situ hybridization of EBER. EBV infection may be related to the pathogenesis of primary SCC. Further evidence and studies are required to establish optimal strategy for this rare disease.

## Background

Primary squamous cell carcinoma (SCC) of stomach is extremely rare [1–3]. To date, only approximately 100 cases have been reported in the literature [4]. Pathogenesis remains unclear, and there is only one case report which suggests that Epstein Barr virus (EBV) infection may be associated to the pathogenesis of primary SCC of stomach. Herein, we report a case of EBV infection-associated primary SCC of stomach, which was treated with radical resection and adjuvant chemotherapy.

## Case presentation

**Fig. 1:**
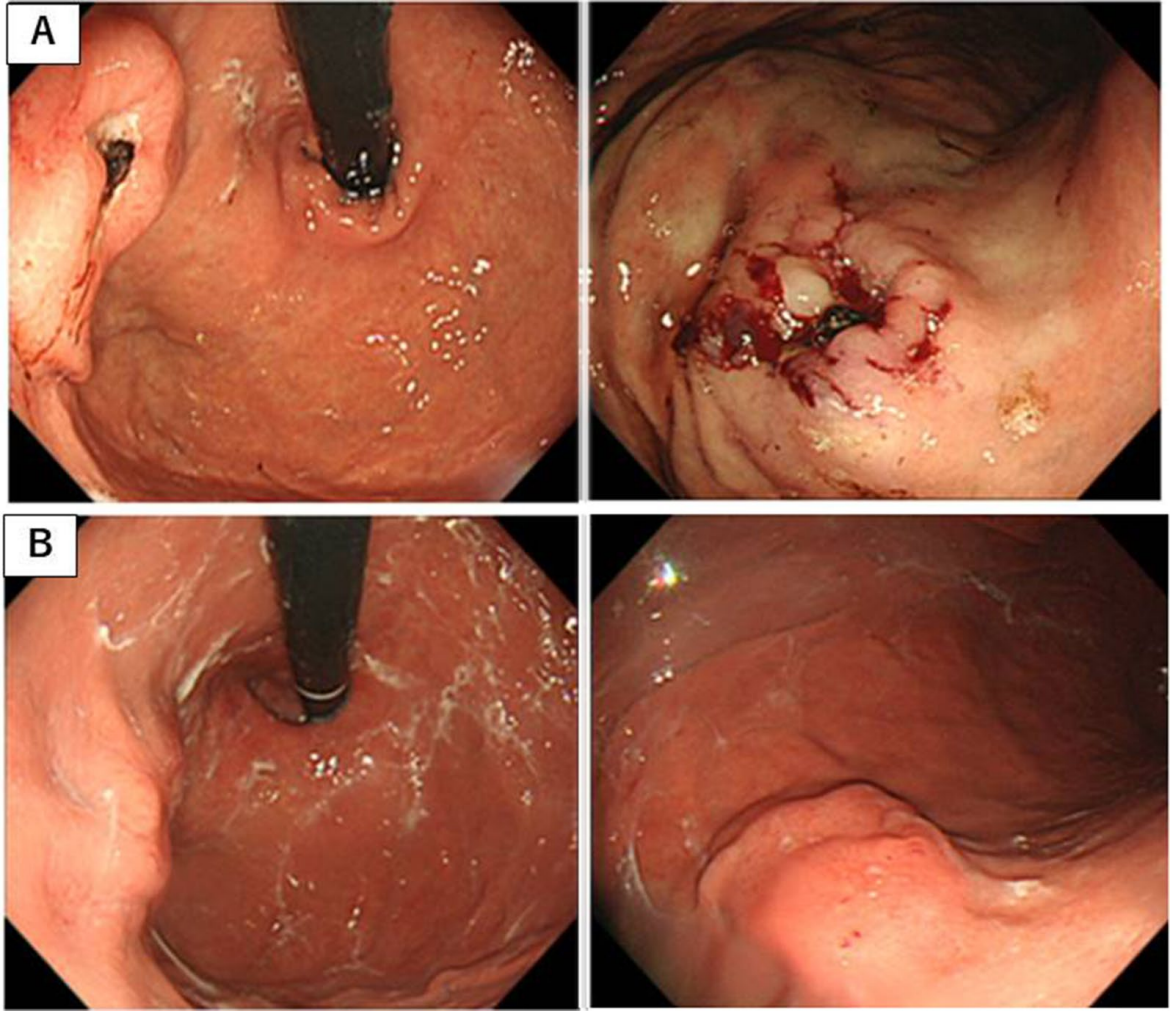
Esophagogastroduodenoscopy (EGD) findings. **A** Initial EGD revealed a bleeding gastric ulcer in the upper part of gastric corpus. **B** Follow-up EGD after 3 months revealed submucosal tumor-like protrusion instead of ulcer

**Fig. 2:**
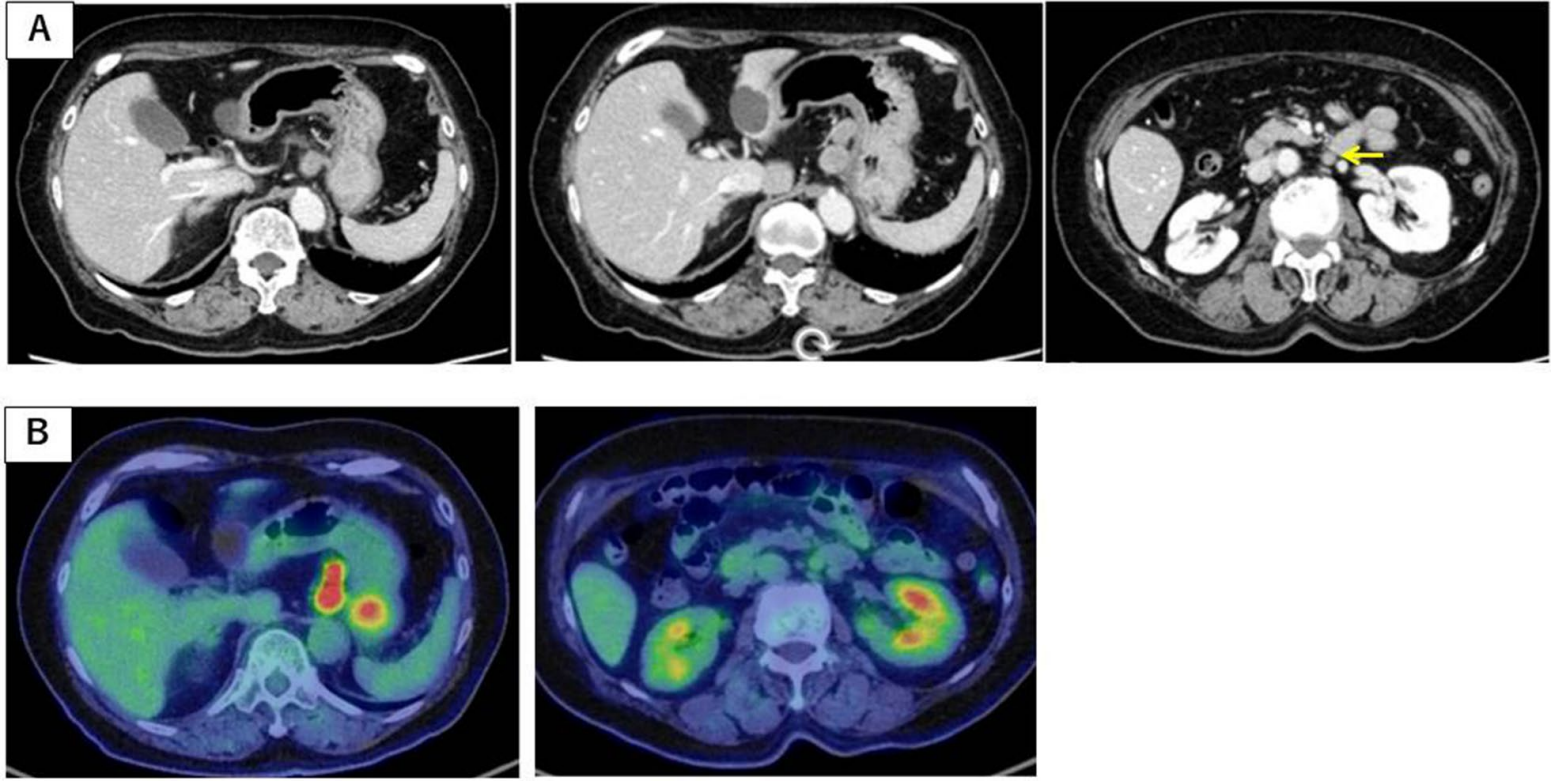
Computed tomography (CT) findings and 18F-fluorodeoxyglucose positron emission tomography (^18^F-FDG PET) CT imaging findings. **A** CT showed a mass approximately 3 cm in diameter in the gastric cardia, swollen lymph nodes along with the lesser curvature and para-aortic lymph node (arrow). **B** ^18^F-FDG PET/CT showed strong uptake of FDG in the lymph nodes of lesser curvature, while para-aortic lymph node indicated weak uptake

A 70-year-old woman was presented to referring hospital with hematemesis. Emergency esophagogastroduodenoscopy (EGD) revealed a bleeding gastric ulcer in the upper part of gastric corpus (Fig. 1A) and the coagulation therapy was followed. After a 3-month follow-up, EGD revealed a submucosal tumor-like protrusion instead of an ulcer (Fig. 1B). Abdominal computed tomography revealed a mass in the upper part of stomach and swollen lymph nodes along with the lesser curvature and para-aortic lymph node (Fig. 2A). No distant metastasis or ascites were identified. ^[18]^F-FDG PET/CT imaging demonstrated strong FDG uptake in a mass of stomach and the lymph nodes of lesser curvature, otherwise uptake of FDG in para-aortic lymph node was weak (Fig. 2B). Biopsy could not confirm the definitive diagnosis. For the definitive diagnosis, we performed a total gastrectomy, cholecystectomy with para-aortic lymph node sampling and Roux-en-Y reconstruction. The postoperative course was uneventful and the patient was discharged on day 11 after the operation.

**Fig. 3:**
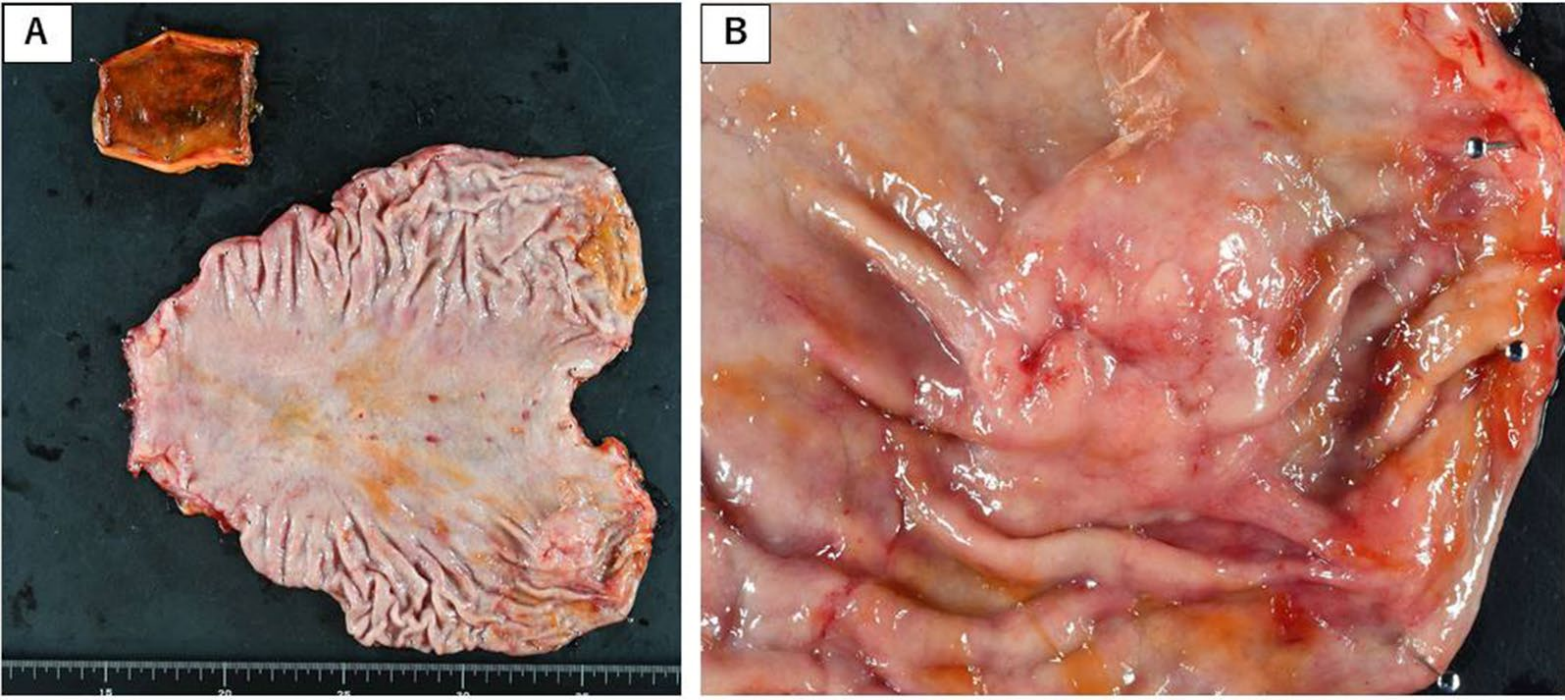
**A** Surgically resected specimen. **B** Macroscopic observation indicated a protruding mass in the gastric cardia along the posterior wall

The macroscopic examination of the resected specimen revealed a protruding mass, measuring 38 × 24 mm, in the gastric cardia along the posterior wall (Fig. 3).

**Fig. 4:**
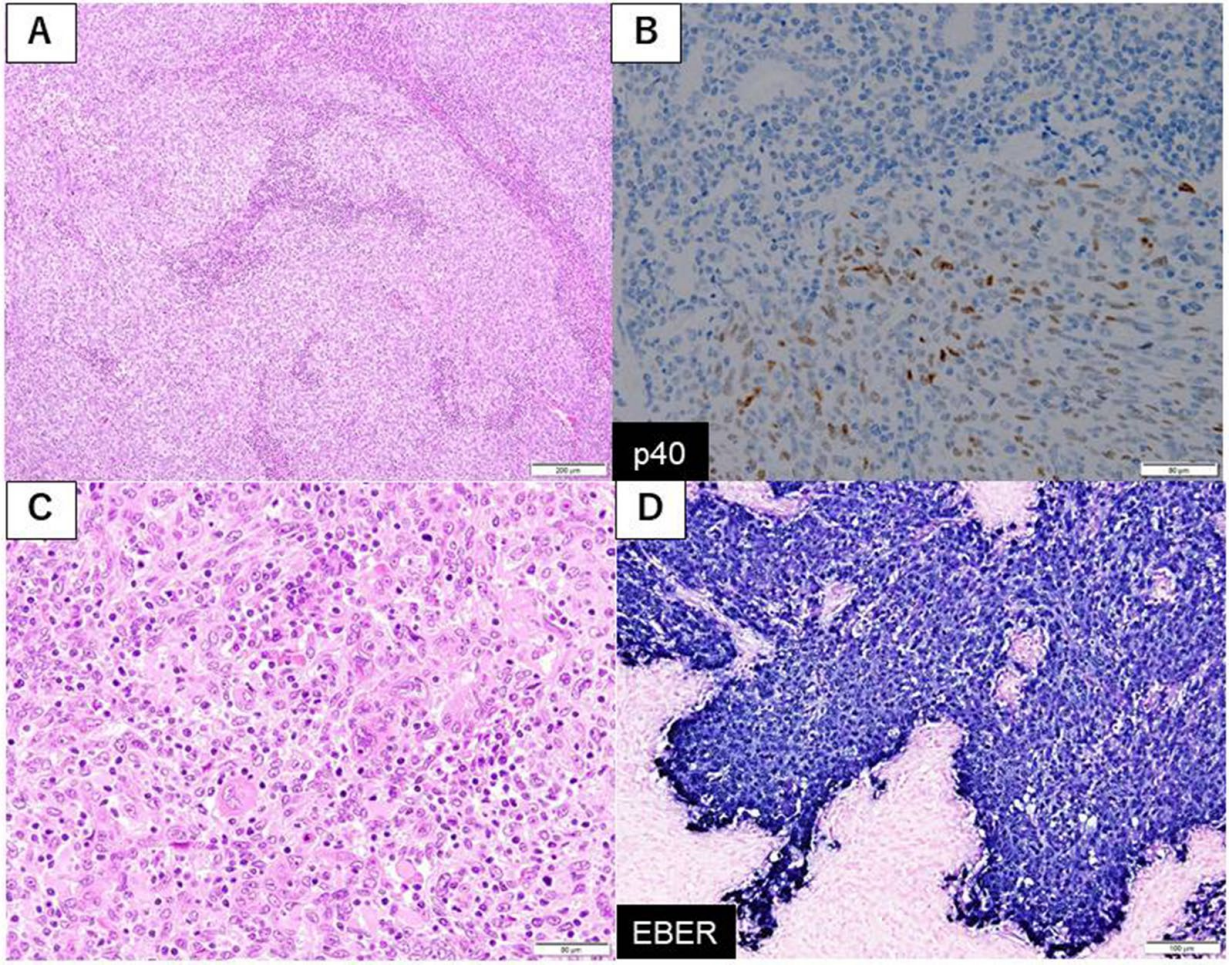
Histopathological findings of resected specimen. **A** The tumor invaded into subserosa forming tumor nests and infiltration of lymphocytes and plasma cells to stroma was found (hematoxylin and eosin, magnification, ×). **B** Immunohistochemistry of p40. **C** Poorly differentiated SCC, exhibiting partial keratinization in the tumor cells (hematoxylin and eosin, magnification, ×). **D** Tumor cells were EBER positive according to in situ hybridization

The histologic findings of the resected specimen stained with hematoxylin and eosin revealed a poorly differentiated SCC with keratin pearl formation cells (Fig. 4A). Immunohistochemistry revealed p40 positivity in tumor cells (Fig. 4B). There were no gland formation and mucus production. Lymphocytes and plasma cells were infiltrated in the tumor stroma and follicle formations were also detected (Fig. 4C). Tumor cells were EBER positive according to in situ hybridization (Fig. 4D). The tumor had not invaded the mucosa of esophagogastric junction. The pathological diagnosis was squamous cell carcinoma with EBV infection of stomach. Nodal metastasis was positive in lesser curvature, but para-aortic lymph nodes were negative. The pathological stage was stage IIB (pT3N1M0), according to the 15th edition of the Japanese Classification of Gastric Carcinoma.

Screening of the head and neck cancer was negative after the surgery. Therefore, we diagnosed this case as EBV infection-associated primary squamous cell carcinoma of the stomach.

Adjuvant chemotherapy with CapeOX was initiated after surgery. At 7 months after surgery, she remains alive without recurrence.

## Discussion

Primary SCC of stomach is extremely rare, with an annual incidence rate of 0.04–0.07% of all gastric cancers [1–3]. It occurs mostly in men and the male/female ratio is 5:1 [3, 5, 6], and the primary lesions were mostly found in the upper-third of the stomach [3].

Primary SCC of stomach is defined according to the following diagnostic criteria proposed by the Japanese Classification of Gastric Carcinoma [7]: (1) all tumor cells are composed by SCC cells, with no adenocarcinomatous components in any sections. (2) Distinct evidence that SCC originates in the gastric mucosa. There are also histopathologic criteria for diagnosis of SCC described by Boswell and Helwig as follows [8]: (1) keratinized cell masses forming keratin pearls. (2) Mosaic cell arrangement. (3) Intercellular bridges. (4) High concentration of sulfhydryl and/or disulfide groups, indicating the presence of keratin and prekeratin. At least one of these criteria should be satisfied for the confirmation of diagnosis.

The immunohistochemistry of CK5/6 and p40 [9] is also effective in the diagnosis of SCC.

Primary SCC has an aggressive behavior, such as metastasizes to lymph node, liver and other organs, they are usually diagnosed at advanced stage, which leads to poor prognosis [10–13]. The standard treatment for the primary SCC of stomach is radical resection [14]. Surgery alone might be insufficient for in advanced cases, therefore various adjuvant chemotherapy regimens were administered. However, most results were not promising and optimal regimen for the primary SCC of stomach has not yet been established [2, 3, 6, 14].

Although the pathogenesis of the primary SCC of stomach is still unknown, several hypotheses concerning the origin of the SCC have been reported as follows [1, 3, 4, 6, 15, 16]: (1) squamous differentiation in a preexisting adenocarcinoma; (2) squamous metaplasia of the gastric mucosa; (3) nests of ectopic squamous epithelium in the gastric mucosa; (4) multipotent stem cells in the gastric mucosa which has a potential to differentiate any type of cell. Takita et al. reported that EBV infection may be related to the pathogenesis of at least some primary SCC of stomach [17]. In this study, a liquid hybridization assay for HPV infection and a polymerase chain reaction for EBV infection was performed and revealed the presence of EBV infection in surgical specimens of the tumor.

EBV infection is known to cause gastric cancer, and EBV infection-associated gastric cancer accounts for approximately 10% of all gastric cancer [18]. EBV infection-associated gastric cancer is defined by monoclonal proliferation of cancer cells with latent EBV infection, as demonstrated by EBV-encoded small RNA (EBER) in situ hybridization [19].

A histological feature of EBV infection-associated gastric cancer is a mainly diffuse-type carcinoma accompanied by abundant lymphocyte infiltration. In case of the tumor invading submucosa, undifferentiated cancer cells and infiltrated lymphocytes compose a mass, causing submucosal nodules [20].

Recent cancer genome atlas research has divided gastric cancer into four molecular subtypes as follows [21]: (1) tumors positive for EBV; (2) microsatellite instability; (3) chromosome instability; (4) genomically stable tumors. The detailed mechanisms of how EBV causes gastric cancer are still unknown. It has been reported that EBV nuclear antigen 1 (EBNA1) destabilizes the p53 gene to suppress p-53-mediated apoptosis [22, 23], and also EBER-1 induces the expression of insulin-like growth factor-1 (IGF1), which causes the autocrine action, promoting proliferation of the EBV-infected cancer cells [18, 24].

In our case, histopathological findings revealed the partially keratinization in the tumor cells without gland formation or mucus production, indicating poorly differentiated SCC. Also immunohistochemistry staining of p40 and CK5/6 was positive. There was no squamous metaplasia or ectopic squamous epithelium around the tumor. Tumor cells invaded into subserosa and infiltration of lymphocytes and plasma cells into the stroma was found. All tumor cells were positive for EBER by in situ hybridization. Therefore, we diagnosed this case as EBV infection-associated primary squamous cell carcinoma of stomach. EBV infection-associated primary SCC of the stomach is very rare, and to our knowledge, there have been no other reports ever before which revealed the EBV infection using in situ hybridization. We performed radical resection, but considering lymph node metastasis in resected specimen, adjuvant chemotherapy was required. A standard chemotherapy regimen for this disease has not been established; the choice of its treatment tends to follow the principle of the treatment against gastric adenocarcinoma. On the basis of adjuvant regimen of gastric adenocarcinoma and the regimen against SCC of esophagus, we selected CapeOX. EBV infection-associated gastric cancer has molecular biological characteristics such as DNA methylation and PD-L1/2 overexpression, demethylating agents or anti-PD-L1 antibody may be one of the treatment options in the future. We need further research in this tumor and further studies would benefit the patients affected by this rare disease.

## Conclusion

In conclusion, here we described a case of EBV infection-associated primary SCC of stomach. Adjuvant chemotherapy following radical resection leads to favorable outcomes. Further evidence and studies are required to establish optimal strategy for EBV infection-associated primary SCC of the stomach.

## Data Availability

All data generated or analyzed during this study are included in the published article.
